# Impact of COVID-19 restrictions on healthcare delivery for thalassemia major patients: A perspective from Pakistan

**DOI:** 10.1016/j.amsu.2022.104636

**Published:** 2022-09-09

**Authors:** Mohammad Ebad Ur Rehman, Beenish Sabir, Faizan Fazal, Junaid Tanveer, Tehseen Haider, Abdul Rauf Khalid, Sajeel Saeed, Jawad Basit

**Affiliations:** Department of Medicine, Rawalpindi Medical University, Block E Satellite Town, Rawalpindi, Pakistan

**Keywords:** Healthcare, Thalassemia major

To the Editor,

Thalassemia major belongs to inherited disorders of beta-globin chains of hemoglobin due to defects in β-Globin Genes (HBB), which are clinically significant. The reduced production of β-Globin chains leads to severe iron-loading anemia due to the detrimental state of ineffective erythropoiesis. Thalassemia patients suffer from compromised immunity with increased risks of developing bacterial infection followed by life-threatening sepsis [1]. The cases of thalassemia are evident in almost every geographic location and every ethnic group, with a higher incidence in the Middle East and Asian Subcontinent, including Pakistan, the Mediterranean basin, and tropical and subtropical regions of Africa [2,3]. β-thalassemia major patients require regular blood transfusions to increase their life expectancy with iron chelation therapy and medications; without these, they may die even before adolescence.

Severe Acute Respiratory Syndrome causing Coronavirus-2 (SARS-CoV-2) was isolated from human respiratory epithelial cells, initially outbreak as idiopathic pneumonia in Wuhan, China, and later declared a global pandemic in March 2020 by World Health Organization. The world has paid a huge cost in terms of millions of lives, economic repercussions, social destabilization, and a huge shock to public health systems due to this pandemic [4]. From regional to global, local to national, economy to the health care system, Covid-19 has challenged us to prepare, respond, and control rapid virus transmission [5]. Moreover, this pandemic has unveiled the limitations of even some highly-ranked health care systems regarding resilience.

Moving towards the matter of discussion, Covid-19 lockdown and restrictions have significantly uprooted the balance between blood demand and provision in the blood banks for transfusion-dependent BTM patients [6]. Several countries have reported a reduction in blood collection and supplies by 40%–67% in the blood banks due to restrictions imposed during the pandemic, and Pakistan was one of them [7]. The regular voluntary blood donors showed resistance toward blood donations on one side in fear of getting infected. In contrast, the limitations of setting blood camps into various institutions and venues played a vital role in reducing blood donation practices during a pandemic. Organizations or societies that conduct the blood camps were also hesitant to host blood drives due to fear of the massive community spread of the virus [7]. The rapid transmission rate of SARS-Cov-2 alarmed people to stay isolated and prevent public gatherings. It significantly affected the blood drives, including blood donation and blood provision to deserving β-thalassemia major patients [8].

The pandemic has adversely affected several healthcare services in Pakistan, and consistent services to thalassemia patients are no exception. In Pakistan, thalassemia patients' conditions were worrisome due to depleted blood bank resources during the pandemic. The already-fragmented blood transfusion services faced a severe blow during the pandemic, leading the lives of transfusion-dependent thalassemia patients at stake [9]. The average lifespan of BTM (beta thalassemia major) patients is less than ten years, which is smaller than the average life span of 10–50 years across the globe [10]. Where the thalassemia patients moved to seek regular healthcare services, including drug therapies and blood transfusions, to add another day to their lives, the transport restrictions and fear of getting infected halted the process entirely. During the pandemic and strict lockdown, these patients lacked their approach to blood transfusions and other drug therapies. There were several causes behind it. The conduction of fewer blood drives and peoples' fear of blood donation during pandemic has dramatically limited the availability of blood for thalassemia patients. All the educational institutions were locked down during the pandemic, limiting the ways to conduct blood drives to motivate young students to donate blood. These blood drives have a significant contribution towards transfusion therapies for BTM patients. Moreover, there was an additional burden on the already-limited Pakistani healthcare system of bed-ridden infected patients in Intensive Care Units (ICUs). Studies have reported that 13.4% of COVID-infected patients demand blood transfusion compared to non-infected patients [11]. The scarce availability of blood in banks and increased demand for critical COVID patients have affected adequate blood provision to transfusion-dependent BTM patients. The pandemic has also affected people financially, rendering them jobless, and major thalassemia patients with low socioeconomic status couldn't seek treatment due to their limited resources. In low and middle-income countries, the expensive treatment of BTM leads to 50,000 to 100,000 deaths annually [12].

BTM patients have been inflicted severely during the pandemic due to the health care department's shortfall in managing blood transfusions for these patients. Therefore, in order to prevent such an unfortunate series of events from reoccurring and to deal with the issues efficiently in any future pandemic, the health care departments and the government should plan strategically ahead of time. It is necessary for the blood banks to manage the blood inventory. Moreover, the capacity of blood centers should be increased and improved, a collaboration of work of the blood bank workers and the clinical staff should be there to provide adequate service on appropriate times, first in first out policy should be observed, to avoid wastage of any amount of blood that can be lifesaving for patients. On top of that, there should be well-equipped transfusion services and blood screening facilities available for the patient and donor which should ensure the safety of both. Furthermore, the Public should be made aware of the benefits of donating blood regularly for their health and the impact it can make for the one in need. Accompanying this, the blood banks should maintain proper records of voluntary donors for any critical situation like the pandemic, which was recorded to be a significant source of transfusions in the past. Moreover, to avoid any risk of virus spread, proper blood screening and strict adherence to transfusion triggers should be there. Keeping in mind the safety concerns of donors and the patients, at-home blood transfusion services should be provided. Along with that, managing and monitoring the conditions of BTM patients through telemedicine should be made essential for timely therapy and avoiding social interaction that can put them at risk of virus.

## Ethical approval

Not applicable.

## Sources of funding

None.

## Author contributions

**Mohammad Ebad Ur Rehman**: Study conception, write-up, critical review and approval of the final version, **Beenish Sabir**:Study conception, critical review and approval of the final version, **Faizan Fazal**: Study conception, write-up, critical review and approval of the final version, **Junaid Tanveer**: Study conception, critical review and approval of the final version, **Tehseen Haider**: Study conception, write-up, critical review and approval of the final version, **Abdul Rauf Khalid**: Study conception, write-up, critical review and approval of the final version, **Sajeel Saeed:** Study conception, write-up, critical review and approval of the final version, **Jawad Basit:** Study conception, write-up, critical review and approval of the final version.

## Registration of research studies

1. Name of the registry: Not applicable
2. Unique Identifying number or registration ID: Not applicable
3. Hyperlink to your specific registration (must be publicly accessible and will be checked): Not applicable

## Guarantor

Mohammad Ebad Ur Rehman.

Faizan Fazal.

## Consent

Not applicable.

## Declaration of competing interest

The authors have no conflicts of interest.
